# Octreotide and Pasireotide Combination Treatment in Somatotroph Tumor Cells: Predominant Role of SST_2_ in Mediating Ligand Effects

**DOI:** 10.3390/cancers13081816

**Published:** 2021-04-10

**Authors:** Jessica Amarù, Federica Barbieri, Marica Arvigo, Agnese Solari, Adriana Bajetto, Federica Nista, Claudia Campana, Gabriele Gaggero, Alessandro Prior, Diego Criminelli Rossi, Gianluigi Zona, Diego Ferone, Tullio Florio, Federico Gatto

**Affiliations:** 1 Section of Endocrinology, Department of Internal Medicine, University of Genoa, 16132 Genoa, Italy; je.90@libero.it (J.A.); marica.arvigo@unige.it (M.A.); nistafodi@libero.it (F.N.); c.campana@erasmusmc.nl (C.C.); 2 Section of Pharmacology, Department of Internal Medicine, University of Genoa, 16132 Genoa, Italy; federica.barbieri@unige.it (F.B.); agnese.solari@edu.unige.it (A.S.); adriana.bajetto@unige.it (A.B.); tullio.florio@unige.it (T.F.); 3 Department of Clinical Pathology, IRCCS Ospedale Policlinico San Martino, 16132 Genoa, Italy; gabriele.gaggero@hsanmartino.it; 4 Division of Neurosurgery, Department of Neurosciences Rehabilitation Ophthalmology Genetics Maternal and Child Health, IRCCS Ospedale Policlinico San Martino, 16132 Genoa, Italy; alessandro.prior@hsanmartino.it (A.P.); diego.criminellirossi@hsanmartino.it (D.C.R.); gianluigi.zona@unige.it (G.Z.); 5 Endocrinology Unit, IRCCS Ospedale Policlinico San Martino, 16132 Genoa, Italy; federico.gatto@hsanmartino.it

**Keywords:** GH-secreting pituitary tumors, somatostatin receptor ligands, octreotide, pasireotide, GH4C1 cell line, acromegaly

## Abstract

**Simple Summary:**

First-generation somatostatin receptor ligands, such as octreotide, are the first-line medical therapy in acromegaly. Octreotide shows preferential binding for somatostatin receptor subtype 2 (SST_2_), while the second-generation ligand, pasireotide, has high affinity for multiple SSTs. We aimed to elucidate whether pasireotide acts via other receptors than SST_2_ in somatotroph tumors, and to investigate the potential role of the combination therapy octreotide plus pasireotide. We found that octreotide and pasireotide are superimposable in reducing GH secretion in cultured somatotroph tumor cells, as well as in inhibiting cell proliferation and intracellular pathway activity in rat GH4C1 cells (a model of somatotroph tumors). We did not find any additive/synergistic effect for the combination treatment. Furthermore, we observed that co-incubation with a SST_2_-selective antagonist reversed the inhibitory effect of both compounds. Therefore, the two drugs act mainly via SST_2_ in somatotroph tumor cells, and their combination is not superior to single agent treatment.

**Abstract:**

First-generation somatostatin receptor ligands (fg-SRLs), such as octreotide (OCT), represent the first-line medical therapy in acromegaly. Fg-SRLs show a preferential binding affinity for somatostatin receptor subtype-2 (SST_2_), while the second-generation ligand, pasireotide (PAS), has high affinity for multiple SSTs (SST_5_ > SST_2_ > SST_3_ > SST_1_). Whether PAS acts via SST_2_ in somatotroph tumors, or through other SSTs (e.g., SST_5_), is a matter of debate. In this light, the combined treatment OCT+PAS could result in additive/synergistic effects. We evaluated the efficacy of OCT and PAS (alone and in combination) on growth hormone (GH) secretion in primary cultures from human somatotroph tumors, as well as on cell proliferation, intracellular signaling and receptor trafficking in the rat GH4C1 cell line. The results confirmed the superimposable efficacy of OCT and PAS in reducing GH secretion (primary cultures), cell proliferation, cAMP accumulation and intracellular [Ca^2+^] increase (GH4C1 cells), without any additive effect observed for OCT+PAS. In GH4C1 cells, co-incubation with a SST_2_-selective antagonist reversed the inhibitory effect of OCT and PAS on cell proliferation and cAMP accumulation, while both compounds resulted in a robust internalization of SST_2_ (but not SST_5_). In conclusion, OCT and PAS seem to act mainly through SST_2_ in somatotroph tumor cells in vitro, without inducing any additive/synergistic effect when tested in combination.

## 1. Introduction

Acromegaly is a severe systemic condition mainly due to the presence of a growth hormone (GH)-secreting pituitary tumor (>95% of cases) [1]. GH excess results in high circulating levels of insulin-like growth factor-1 (IGF-1), hence leading to increased patients’ morbidity and mortality [1]. Therefore, a primary aim of acromegaly management is to reach normal age-adjusted IGF-1 levels, as well as safe GH values (defined as random GH <1 µg/L or <2.5 µg/L, according to the sensitivity of the assays) [2,3].

Surgery represents the first-line treatment in most patients, while medical therapy is used in clinical practice when surgery is not feasible (or the patient refuses it), and in case of disease persistence and/or recurrence [4].

First-generation somatostatin receptor ligands (fg-SRLs), octreotide (OCT) and lanreotide (LAN), still represent the first choice as medical treatment in acromegaly patients [2,5,6]. Both OCT and LAN are stable octapeptides showing a preferential binding affinity for the somatostatin receptor subtype 2 (SST_2_), widely expressed in GH-secreting pituitary tumor cells [7,8]. In this context, a number of studies already demonstrated a positive correlation between SST_2_ expression in GH-secreting tumor cells and fg-SRL efficacy in reducing hormone secretion both in vitro and in vivo [9,10,11,12,13,14].

However, despite a relatively high expression of SST_2_ in somatotroph tumors, about half of acromegaly patients are only partially responders (or even completely resistant) to fg-SRLs [15].

More recently, a multi-receptor ligand, pasireotide (PAS), has been synthetized, and it is currently available for clinical use in acromegaly [5,16]. PAS is a cyclohexapeptide with long half-life, showing binding affinity in the nanomolar range for multiple SSTs, particularly SST_5_ and SST_2_ (SST_5_ > SST_2_ > SST_3_ > SST_1_) [7,16].

Nowadays, guidelines and Consensus Statements suggest the use of PAS mainly as second-line medical therapy in acromegaly patients showing partial responses to fg-SRLs [2,5].

Clinical studies, comparing the efficacy of OCT and PAS in unselected patients, proved that PAS is more effective than OCT in normalizing IGF-1 levels, while it is as effective as OCT in restoring safe GH values [17]. In line with this finding, in vitro studies demonstrated that OCT and PAS are equally effective in reducing GH secretion into conditioned media of primary cultures from somatotroph tumor cells [10,18,19]. Furthermore, the inhibitory effect of PAS seems to be directly correlated with the expression of SST_2_ (and not SST_5_) on tumor cells [10,19]. Accordingly, a recent study by Muhammad and colleagues showed that the in vivo effect of PAS in acromegaly patients, partially-responders to fg-SRLs, is mainly mediated by SST_2_ expression [20].

At odds with these findings, other authors supported the hypothesis that SST_5_ plays a predominant role in driving the effects of PAS in somatotroph tumors, based on its peculiar receptor binding affinity [21,22]. Although current evidence suggests that in acromegaly PAS exerts its biological activity via SST_5_ only in tumors showing a negligible amount of SST_2_ (or in SST_2_-negative lesions), the debate is still open [19,20,21].

Furthermore, a number of elegant in vitro studies, mainly carried out in SST-transfected cells, suggested that PAS might act as a biased agonist toward SST_2_, based on the differential modulation of the receptor internalization, trafficking, as well as its peculiar effects on intracellular Ca^2+^ accumulation and ERK1/2 phosphorylation (partial agonist/antagonist), compared to both OCT and naïve somatostatin (SRIF) [23,24,25].

Therefore, moving from the different biological characteristics blamed on PAS, its combination with a SST_2_-preferential compound, such as OCT, could theoretically result in either a synergistic/additive or an antagonistic effect. Otherwise, whether the combination of PAS and OCT was comparable to the use of each single compound, this finding would suggest that the two drugs act on convergent pathways.

In this complex scenario, we investigated the effect of combined treatment with OCT and PAS on GH secretion from somatotroph cells (primary cell cultures from human pituitary tumors), in comparison with single treatments. The inhibitory effect of the two compounds, alone and in combination, on the in vitro GH release was correlated to the expression of SST_2_ and SST_5_ on tumor samples.

Furthermore, to better elucidate the intracellular mechanisms underlying the biological activity of OCT, PAS and their combination, rat GH4C1 cell line was used as a model of somatotroph tumors [26]. In this setting, we investigated the effects of the two SRLs on cell proliferation, as well as intracellular calcium concentration and cAMP synthesis, which represent the main signaling pathways affected by SSTs to modulate hormonal release in neuroendocrine cells [27,28]. Finally, the co-incubation with the selective SST_2_ antagonist, BIM-23627, served to point out the specific role of SST_2_-targeting in the observed responses.

## 2. Materials and Methods

### 2.1. Reagents

The SST_2_-preferential compound octreotide (OCT) and the multi-receptor ligand pasireotide (PAS) were obtained from Novartis Pharma A.G. (Basel, Switzerland). The SST_2_ antagonist BIM-23627 was kindly provided by Biomeasure Incorporated/IPSEN (Milford, MA, USA).

### 2.2. Patients and Tumors

Eleven acromegaly patients who underwent adenomectomy by trans-sphenoidal surgery at the Neurosurgery Unit of our center (IRCCS Ospedale Policlinico San Martino, Genoa, Italy) were included in the study.

Inclusion criteria of the study were: (1) availability of enough viable cells to establish a primary culture; (2) adequate cell number to test in the same experiment, at least in triplicate, the antisecretory effect of 72-h OCT and PAS treatment (alone and in combination); and (3) availability of paraffin-embedded tumor tissues in order to perform immunohistochemistry for both SST_2_ and SST_5_.

Of note, tumor samples from patients which underwent radiotherapy before adenomectomy were not considered eligible for the study. No other exclusion criteria, based on patient or tumor characteristics, were applied.

The diagnosis of acromegaly was based on clinical features, biochemical evidence of GH hypersecretion (lack of suppression of GH to <1 µg/L after a 2-h oral glucose tolerance test), IGF-1 levels above the age-adjusted upper limit of normality range (>1 xULN), and the presence of a pituitary adenoma detected by magnetic resonance imaging (MRI).

General, clinical and biochemical characteristics of the patients, as well as histopathological information of the tumors are reported in Table 1.

Briefly, four patients (36.4%) were females and seven males (63.6%), median age at time of surgery was 44.1 years (range 16–57). The majority of patients had a macroadenoma (9/11, 81.8%), although most lesions showed low proliferation index (9 out of 11 cases, according to the definition of Trouillas and colleagues [29]). Indeed, based on pathology evaluation, only patients n. 8 and n. 10 had an aggressive lesion (Table 1).

Three patients (27.3%) received pre-surgical medical treatment with fg-SRLs for at least three months before surgery (octreotide LAR or lanreotide Autogel) (Table 1). Although they did not reach IGF-1 normalization (≤1 xULN), patient n.1 and patient n.2 showed IGF-1 reduction of 68% and 48%, respectively. As for patient n.8, he was referred to our center after starting pre-surgical SRL treatment, and IGF-1 values at diagnosis were not available.

At the last biochemical evaluation before adenomectomy, GH levels were 18.72 ± 19.19 µg/L (mean ± SD, median 11.80 µg/L), while IGF-1 values (expressed as ratio to the age-adjusted upper limit of normality, ULN) were 2.29 ± 0.88 xULN (mean ± SD, median 2.02 xULN). Absolute IGF-1 values were 593.5 ± 202.9 µg/L (mean ± SD, median 509.0 µg/L). GH and IGF-1 values were significantly and directly correlated (Pearson’s r = 0.869, *p* = 0.005).

After neurosurgery, four patients needed adjuvant SRL treatment (patients n. 1, 2, 5 and 6). Mean IGF-1 reduction at the last follow-up was 40.8% (±29.6, median 44.7%), with three subjects reaching biochemical control (IGF-1 ≤1 xULN; patients n. 1, 2 and 6). Of note, due to incomplete response to SRL therapy, patient n. 5 underwent following medical treatment with the GH-receptor antagonist pegvisomant.

Informed consent was acquired from all subjects involved in the study after the approval of the Institutional Ethical Committee (IEC, CER Liguria register number: 360/2019).

### 2.3. Immunohistochemistry (IHC)

SST protein expression was evaluated in formalin-fixed paraffin-embedded tumor samples (5 µm-thick sections) using the Dako EnVision^®^+ Dual Link System-HRP (DAB+) kit (Dako/Agilent, Santa Clara, CA, USA) as previously reported [13]. To detect SST_2_ and SST_5_, 1:200 dilution of rabbit anti-SST_2_ (RRID: AB_2737601) and rabbit anti-SST_5_ (RRID: AB_10859946) monoclonal antibodies (UMB-1 and UMB-4, respectively, Abcam, Cambridge, UK) was used. Receptor staining on tumor samples was semiquantitatively scored by using an immunoreactivity scoring system (IRS). IRS is calculated by the product of the percentage of positive cells (4, >80%; 3, 51% to 80%; 2, 10% to 50%; 1, <10%; 0, 0%) and the intensity of the staining (3, strong; 2, moderate; 1, mild; 0, no staining), which results in IRS scores between 0 (no staining) and 12 (maximum staining) [13].

### 2.4. Cell Cultures

#### 2.4.1. Primary Cell Cultures of Somatotroph Tumors

Primary cultures were set according to a protocol developed in our laboratory. In detail, immediately after surgery, a fragment of the resected fresh tumor was mechanically dissociated under sterile conditions to obtain a single-cell suspension. Cells were filtered through a 70 µm cell strainer and then treated with deionized water for a few seconds to remove red blood cells. The resulting cells were cultured at 37 °C in a humidified CO_2_ incubator in 25 cm^2^ flask in Dulbecco Modified Eagles Medium (D-MEM, Sigma-Aldrich, St. Louis, MI, USA), supplemented with 10% fetal bovine serum (FBS), 1% non-essential amino acids, 1% penicillin-streptomycin and 1% L-glutamine (all from Euroclone S.p.A., Milan, Italy).

#### 2.4.2. GH4C1 Cell Line

Rat somatotroph GH4C1 cells (RRID:CVCL_0276) were purchased from ATCC (ATCC^®^ CCL-82.2™) and routinely grown at 37 °C in a humidified CO_2_ incubator in D-MEM/Nutrient Mixture F-12 HAM (Sigma-Aldrich, St. Louis, MN, USA), supplemented with 10% FBS, 1% non-essential amino acids, 1% penicillin-streptomycin and 1% L-glutamine (Euroclone S.p.A., Milan, Italy) [30].

### 2.5. In Vitro GH Secretion

Cells from pituitary tumor primary cultures were plated in 48-well plates at a density of 2 × 10^5^ cells/well and incubated for 72 h with or without OCT and PAS (alone and in combination), at the concentration of 10^−8^ M, in triplicate.

GH4C1 cells (2 × 10^5^ cells/well) were seeded in 48-well plates in complete medium with 10% FBS and incubated at 37 °C in a humidified 5% CO_2_ atmosphere for 24 h. The medium was replaced by adding serum-free D-MEM/Nutrient Mixture F-12 HAM (0.1% BSA) for 24 h. Cells were then treated with 10^−8^ M of OCT and PAS, alone or in combination, and incubated for a further 6 and 24 h, in triplicate. At the end of the incubation times, conditioned media from cell cultures were centrifuged at 600× *g* for 5 min and stored at −20 °C until GH measurement.

GH concentration in culture media from primary cell cultures and GH4C1 cells was measured by hGH (RRID: AB_2813811) or m/rGH (RRID: AB_2813812) ELISA (Mediagnost, Reutlingen, Germany), respectively. Intra- and inter-assay coefficients of variation were 5.46% and 4.34% for hGH ELISA Kit or <5.0 and <10% for m/rGH ELISA Kit, respectively.

### 2.6. Cell Proliferation

GH4C1 cells (3 × 10^4^ cells/well) were seeded in 96-well plates in a final volume of 200 µL and incubated at 37 °C in a humidified 5% CO_2_ atmosphere for 24 h. Culture medium was then replaced by 180 µL of serum-free D-MEM/Nutrient Mixture F-12 HAM containing 0.1% BSA, and cells were incubated for a further 24 h. OCT, PAS and BIM-23627 (10^−8^ M), alone or in equimolar combination, were added in a final volume of 200 µL per well, and cells were incubated for a further 48 h in triplicate. Cell proliferation was evaluated using the BrdU Cell Proliferation Assay Kit (Cell Signaling Technology, Danvers, MA, USA) according to the manufacturer’s instructions. Results were obtained by determining the mean value of four different experiments in triplicate.

### 2.7. Quantification of cAMP Levels

GH4C1 cells (6 × 10^5^ cells/well) were plated in 6-well plates, allowed to attach for 24 h and starved for a further 24 h in serum-free medium. Then, cells were treated with OCT (10^−8^ M), PAS (10^−8^ M), and BIM-23627 (10^−8^ M) for 1 h, in the presence of 1 µM 3-isobutyl-1-methylxanthine (IBMX) as phosphodiesterase inhibitor. Cell lysis and cAMP quantitative determination were carried out using the Parameter™ mouse/rat cAMP kit (R&D Systems, Minneapolis, MN, USA) following the manufacturer’s instructions, as reported in [31]. The results are expressed as the means ± SEM of triplicate samples from three independent experiments.

### 2.8. Intracellular [Ca^2+^] Measurement

GH4C1 cells (2 × 10^5^ cells/well) were plated in 35 mm Cell Imaging dishes (Eppendorf, Milan, Italy), coated with poly-L-lysine (10 µg/mL). After 24 h, cells were washed with PBS and loaded with Fura-2 AM (2 µM) (Abcam, Cambridge, UK) in Locke buffer (HEPES 10 mM pH 7.4, NaCl 150 mM, KCl 5.5 mM, CaCl_2_ 1.5 mM, MgSO_4_ 1.2 mM, glucose 10 mM) for 20 min at room temperature to avoid intracellular compartmentalization [30] and then washed for 10 min with the same balanced salt solution buffer. After 5 min OCT (10^−8^ M) and PAS (10^−8^ M) were added, and then the Ca^2+^ influx was induced by perfusing cells with KCl 40 mM [32].

The emission of fluorescence was recorded with a digital video camera connected to an image recording system (FAB Crea, Genoa, Italy). Then Ca^2+^ images were processed with ImageJ Fiji software (ROI tool). Data were calculated as 340/380 nm ratio values from the analysis of 20 cells per field, from five independent experiments.

### 2.9. Immunocytofluorescence (IF)

GH4C1 cells (2 × 10^4^ cells/well) were grown on 8-well chamber-slides (BD Bioscience, San Jose, CA, USA) at 37 °C in 5% CO_2_ for 48 h and starved for 24 h in serum-free medium. Cells were stained with Vybrant™ DiI Cell-Labeling Solution (Invitrogen, Carlsbad, CA, USA) then incubated with OCT or PAS (10^−7^ M) for 20 min, rinsed with PBS and fixed with 4% paraformaldehyde for 10 min. Slides were processed as previously described [33] and incubated with rabbit monoclonal SST_2_ and SST_5_ antibodies (both from Abcam, Cambridge, UK), at room temperature for 1 h (1:200 dilution) [34]. Fluorochrome-conjugated antibody (goat anti-rabbit Alexa Fluor-488; RRID: AB_2633280; Invitrogen, Carlsbad, CA, USA) was then applied. Negative controls omitting primary antibodies were included in all the experiments. Coverslips were mounted with ProLongTM Gold Antifade Mountant (ThermoFisher Scientific, Waltham, MA, USA). Slides were photographed with a confocal microscope (BioRad MRC 1024 ES).

### 2.10. RNA Isolation and Quantitative Real Time (qRT)-PCR

Total RNA was extracted from GH4C1 cells using the Aurum™ total RNA Mini Kit and reverse transcribed using iScript cDNA Synthesis Kit (both from BioRad, Hercules, CA, USA). cDNA was amplified using the BioRad’s SsoFast™ EvaGreen supermix on a CFX96 RT-PCR Detection System (BioRad, Hercules, CA, USA). Published forward and reverse primer sequences for rat SST_1–5_ subtypes [35], as well as housekeeping genes, were synthesized by TIB MolBiol (Genoa, Italy). Levels of target genes in each sample were normalized on the basis of housekeeping gene amplification and reported as relative values [36]. All RT-PCR runs included negative controls without mRNA templates and cDNA transcription to check reagents for contaminations.

### 2.11. Western Blotting

GH4C1 cells (5 × 10^6^ cells) were seeded in 150 cm^2^ flasks in complete medium. At confluence, cells were solubilized for membrane protein extraction and protein content was detected by immunoblot analysis, as previously described [37]. To evaluate SST protein expression in GH4C1 cells, the following rabbit polyclonal antibodies were used: anti-SST_1_, anti-SST_2_ and anti-SST_5_ (RRID: AB_2196045, RRID: AB_2255396 and RRID: AB_2196380, respectively; Santa Cruz Biotechnology, Inc., Dallas, TX, USA) using a 1:500 dilution. β-actin was detected by incubation with an HRP-conjugated anti-β-actin antibody (RRID: AB_2714189; Santa Cruz, Biotechnology, Inc., Dallas, TX, USA; dilution 1:10,000). Protein detection was assessed by ECL detection system (GE Healthcare, Chicago, IL, USA) and analyzed using a dedicated chemiluminescence imaging system (UVITEC Alliance, UVITEC, Cambridge, UK).

### 2.12. Combination Index Calculation

The interaction between the effect of OCT and PAS was analyzed using the median-effect method [38,39], expressed as combination index (CI). CI describes the nature of drug–drug interactions that can be antagonistic (CI > 1), additive (CI = 1) or synergistic (CI < 1) for various concentrations [40]. CI values were calculated using the CompuSyn software (ComboSyn Inc., Paramus, NJ, USA), following the method by Chou and Talalay [38].

### 2.13. Statistical Analysis

Results are expressed as mean ± standard deviation (SD) when reporting data from patients and related tumor samples, while mean ± standard error (SEM) is used for repeated experiments performed in GH4C1 cell line. Statistical analysis was performed using the GraphPad Prism 6.0 software (GraphPad Software Inc., San Diego, CA, USA). After performing normality test, one-way ANOVA followed by the Newman–Keuls post-hoc test or Kruskal–Wallis test followed by Dunn’s post-hoc test were used to analyze differences between groups. Correlation coefficients were calculated using Pearson’s r (data normally distributed). Statistical significance was established at *p* < 0.05.

## 3. Results

### 3.1. SST Protein Expression in Pituitary Tumor Samples

**Figure 1:**
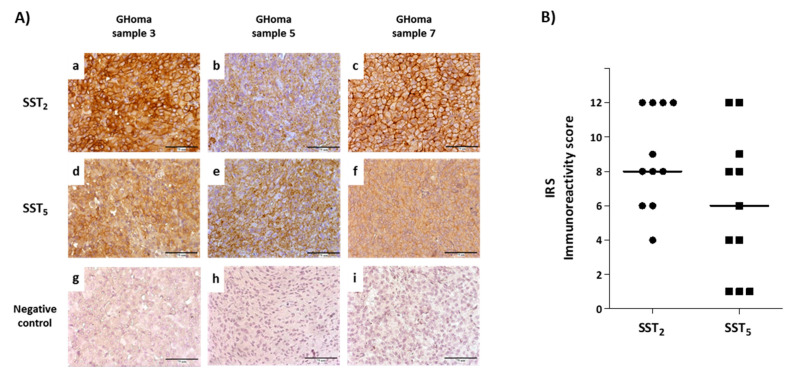
Immunohistochemical evaluation of SST_2_ and SST_5_ in somatotroph pituitary tumors. Panel (**A**)) Representative IHC of SST_2_ and SST_5_ in three different GHomas (scale bar, 75µm): (**a**) high SST_2_ IRS in sample 3 (IRS 12); (**b**,**c**) high-intermediate SST_2_ IRS in samples 5 and 7 (IRS 8); (**d**) high-intermediate SST_5_ IRS in sample 3 (IRS 9); (**e**) high SST_5_ IRS in sample 5 (IRS 12); (**f**) intermediate SST_5_ IRS in sample 7 (IRS 4); (**g**–**i**) negative controls processed with the omission of primary antibodies. Panel (**B**)) Scatter plot of SST_2_ and SST_5_ IRS of all samples (*n* = 11) and median line. Each dot and square in panel (**B**) represents a single patient.

Immunohistochemistry performed on paraffin-embedded tumor sections showed the presence of SST_2_ and SST_5_ in all somatotroph tumor samples analyzed, although with a heterogeneous expression pattern (Figure 1A). We found a median high-intermediate immunoreactivity score (IRS) for SST_2_ (IRS = 8) and intermediate for SST_5_ (IRS = 6) (Figure 1B). In detail, one tumor (patient 8) showed intermediate SST_2_ score (IRS = 4), six tumors (patients 1, 2, 5, 7, 10 and 11) high-intermediate SST_2_ expression (IRS between 6–9), while four samples (patients 3, 4, 6 and 9) had the maximum score (IRS = 12). As far as SST_5_ expression, three tumors (from patients 6, 8 and 11) showed low score (IRS = 1), two samples (patients 7 and 10) had intermediate score (IRS = 4), four (patients 1, 2, 3 and 9) showed high-intermediate SST_5_ expression (IRS between 6 and 9), and two tumors (patients 4 and 5) received the maximum score (IRS = 12).

SST_2_ and SST_5_ IRS showed a slight trend for higher values in tumors from treatment-naïve patients compared to those which underwent SRL therapy before surgery (see Table 1), although this difference was not statistically significant (SST_2_ *p* = 0.21, and SST_5_ *p* = 0.65). No correlation was found between SST_2_ and SST_5_ IRS (r = 0.49, *p* = 0.13).

### 3.2. Effect of OCT and PAS on GH Secretion in Human Somatotroph Cells

**Figure 2:**
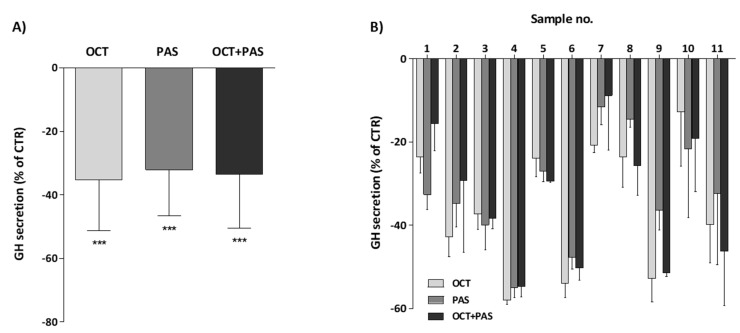
In vitro effect of OCT (10^−8^M) and PAS (10^−8^ M), alone or in combination, on GH secretion in 11 primary cultures from somatotroph tumors. Panel (**A**)) Overall efficacy of OCT, PAS and OCT+PAS after 72 h of treatment. Panel (**B**)) Detailed antisecretory effect of OCT, PAS and OCT+PAS in the different cell cultures. Data are expressed as mean ± SD of each experiment, performed in triplicate, and reported as percent inhibition of control cells. (CTR, control; OCT, octreotide; PAS, pasireotide; ***, *p* < 0.001 vs. control).

The effect of OCT and PAS (used at the concentration of 10^−8^ M) on GH secretion was tested in the primary cultures obtained from all of the eleven somatotroph tumors included in this study. On the whole, OCT and PAS, alone or in combination, significantly reduced GH secretion, compared to untreated cells (OCT: −35.4% ± 11.1, PAS: −32.1% ± 14.4, OCT+PAS: −33.5% ± 17.0; *n* = 11, *p* < 0.001 vs. control). The inhibitory effect of the two drugs was superimposable, and their combination had a similar efficacy compared to each single monotherapy (Figure 2A). Interestingly, dose-response curves (10^−9^ to 10^−7^ M) were also generated from two pituitary primary cultures (patients n. 10 and 11) (Figure S1A) that were analyzed for drug interaction calculating the combination index (CI). The CI value obtained (1.74) was indicative of a moderate antagonistic effect (no synergistic or additive effect).

Focusing on the individual response of each cell culture, OCT displayed a reduction in GH secretion >20% in 10/11 cases, PAS in 9/11 cases and OCT+PAS in 8/11 tumor samples (Figure 2B).

**Figure 3:**
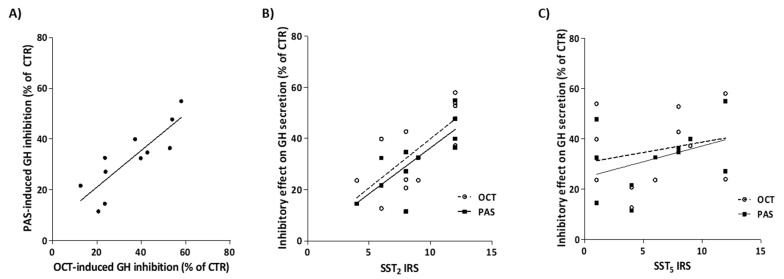
Correlation between the inhibitory OCT and PAS effects on GH secretion and SST_2/5_ protein expression in somatotroph tumors. (**A**) Correlation between OCT- and PAS-induced inhibition of GH secretion in eleven somatotroph tumor cell cultures. Each dot represents a single patient. (**B**,**C**) Correlation between SST_2_ (panel B) or SST_5_ (panel (**C**)) IRS and the inhibitory effect on GH secretion of OCT and PAS in 11 adenoma cell cultures. (CTR, control; OCT, octreotide; PAS, pasireotide; IRS, immunoreactivity score).

Moreover, the inhibition of GH secretion induced by OCT was strongly and directly correlated with the effect induced by PAS (r = 0.85, *p* = 0.001) (Figure 3A). Furthermore, the efficacy of OCT or PAS alone directly correlated with the results observed testing the combination of the two drugs (OCT vs. OCT+PAS: r = 0.90, *p* < 0.001; PAS vs. OCT+PAS: r = 0.81, *p* < 0.01) (Figure S2A,B).

The inhibitory effect of OCT and PAS on GH secretion was significantly and directly correlated with SST_2_ IRS (r = 0.71, *p* < 0.05, and r = 0.78, *p* < 0.01, respectively) (Figure 3B). On the other hand, no significant correlation was found between the efficacy of the two compounds and SST_5_ IRS (OCT: r = 0.22, *p* = 0.52; PAS: r = 0.39, *p* = 0.23) (Figure 3C).

Neither SST_2_ IRS nor SST_5_ IRS were significantly correlated with the effect of OCT+PAS on GH secretion in vitro, although a trend for a direct correlation was observed between the efficacy of the combination therapy and SST_2_ IRS (r = 0.57, *p* = 0.063) (Figure S2C,D).

### 3.3. SST mRNA and Protein Expression in GH4C1 Cells

The rat GH-secreting pituitary adenoma cell line GH4C1 was used as a model to deeper investigate the role of SSTs in mediating OCT and PAS effects (alone and in combination) and the intracellular mechanisms involved.

SST expression was evaluated by both qRT-PCR and Western Blot methods. Of note, SST_1_, SST_2_, as well as SST_5_ mRNA expression was detected in GH4C1 cells. On the other hand, SST_3_ mRNA was not detected in our cells (Figure S3A). Immunoblot analysis of GH4C1 membrane lysates, performed in basal condition, confirmed the results of mRNA evaluation, showing the expression of SST_1_, SST_2_ and SST_5_ in GH4C1 cells, with a 1.5-fold higher expression of SST_2_ compared to both SST_1_ and SST_5_ (Figure S3B).

### 3.4. Inhibitory Effect of SRLs on Cell Proliferation, cAMP and Ca^2+^ Levels in GH4C1 Cells

**Figure 4:**
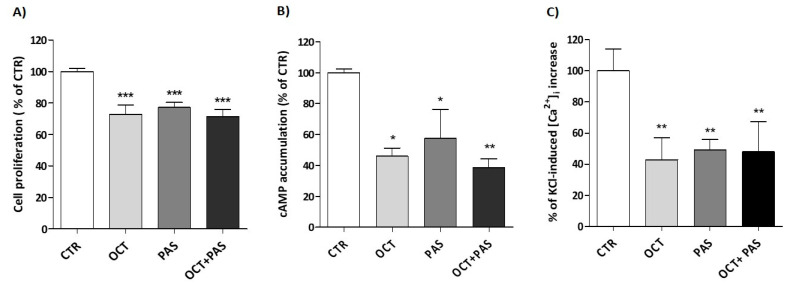
Effect of OCT (10^−8^ M) and PAS (10^−8^ M), alone or in equimolar combination, on cell proliferation, intracellular cAMP and [Ca^2+^]_i_ levels in GH4C1 cells. Panel (**A**)) Antiproliferative effect of SRLs in GH4C1 cell line. Data are expressed as mean ± SEM of four different experiments, performed in triplicate, and reported as percent inhibition of control. Panel (**B**)) Inhibitory effect of OCT, PAS and OCT+PAS on cAMP levels in IBMX-treated GH4C1 cells. Data are expressed as mean percent inhibition of untreated cells ± SEM of three different experiments performed in triplicate. Panel (**C**)) Histograms represent the SRL-induced [Ca^2+^]_i_ decrease in KCl-stimulated cells as mean (%) ± SEM of five independent experiments. (CTR, control; OCT, octreotide; PAS, pasireotide; * *p* < 0.05, ** *p* < 0.01, *** *p* < 0.001 vs. control).

In GH4C1 cells, we observed that OCT, PAS and OCT+PAS (all tested at 10^−8^ M concentration) significantly decrease cell proliferation compared to untreated cells (CTR: 100% ± 2.0, OCT: 72.9% ± 5.8, PAS: 77.1% ± 3.2, OCT+PAS: 71.5% ± 4.6; *p* < 0.001 vs. CTR). Of note, all the treatments showed a comparable efficacy (Figure 4, panel A). The combination OCT+PAS not only did not result in an additive/synergic effect on GH4C1 proliferation, but according to the calculated combination index for the maximal obtained effect (CI = 1.79; dose-response from 10^−10^ to 10^−7^ M), the interaction between the two drugs was moderately antagonistic (Figure S1B).

To assess whether OCT, PAS, and their combination activate different intracellular pathways (or affect the same pathways to a different extent), we evaluated the effects of the differential treatments on cAMP accumulation and intracellular Ca^2+^ levels ([Ca^2+^]_i_).

In detail, both OCT and PAS (10^−8^M concentration) resulted in a significant inhibition of intracellular cAMP levels in IBMX-treated GH4C1 cells (CTR: 100.0% ± 2.5, OCT: 46.0% ± 5.2, PAS: 57.6% ± 18.8; *p* < 0.05 vs. CTR). The combination of OCT+PAS (equimolar concentration 10^−8^M) significantly decreased cAMP accumulation compared to control cells, with an efficacy similar to that observed for the two single drugs (OCT+PAS: 38.6% ± 5.8; *p* < 0.01 vs. CTR) (Figure 4B).

The analysis of [Ca^2+^]_i_ variation in KCl-perfused GH4C1 cells, after 5-min pretreatment with OCT and PAS (10^−8^ M concentration), demonstrated the ability of the two compounds to reduce the depolarization-dependent Ca^2+^ influx (compared to control cells), either when used as single agents or in combination (CTR: 100.0% ± 14.1, OCT: 42.9% ± 14.0, PAS: 49.1% ± 6.8, OCT+PAS: 48.0% ± 19.3; *p* < 0.01 vs. CTR) (Figure 4C). Of note, the efficacy of OCT and PAS in reducing intracellular Ca^2+^ levels, was superimposable, and the combination of the two compounds was comparable to that of single agent treatments (Figure 4C).

### 3.5. Effect of BIM-23627 on SRL-Induced Inhibition of Cell Proliferation and cAMP Accumulation in GH4C1 Cells

To deeply investigate the role of SST_2_ in driving OCT and PAS effects, the SST_2_ antagonist BIM-23627 was used, being tested alone or in combination with both SRLs.

**Figure 5:**
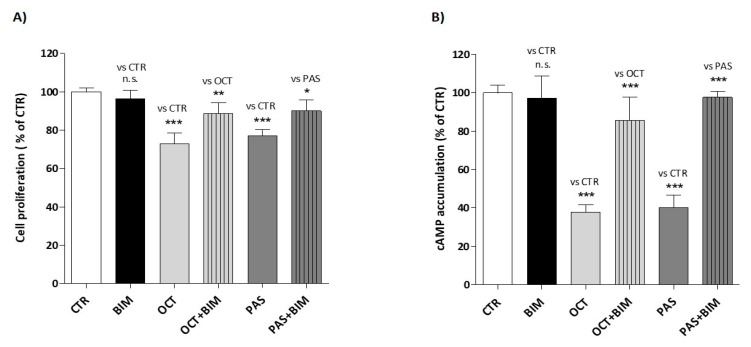
Effect of the SST_2_-selective antagonist (BIM-23627) on SRL-induced inhibition of cell proliferation and cAMP accumulation in GH4C1 cells. Effect of OCT (10^−8^ M), PAS (10^−8^ M) and BIM-23627 (10^−8^ M), alone or in combination, on GH4C1 cell proliferation (**A**) and intracellular cAMP levels (**B**). Data are expressed as mean percent inhibition of untreated cells ± SEM of four different experiments (**A**) and three different experiments (**B**) performed in triplicate. (CTR, control; OCT, octreotide; PAS, pasireotide; BIM, SST_2_-selective antagonist; n.s., not statistically significant; * *p* < 0.05, ** *p* < 0.01, *** *p* < 0.001 vs. control).

As expected, BIM-23627 alone did not affect GH4C1 cell proliferation (10^−8^ M concentration), while it significantly counteracted OCT-induced inhibition of cell proliferation, when tested as equimolar combination (OCT+BIM: 88.7% ± 5.6 vs. CTR, OCT: 72.9% ± 5.8 vs. CTR; OCT+BIM vs. OCT *p* < 0.01) (Figure 5A). Similarly, the equimolar combination of PAS + BIM-23627 resulted in a significant reduction of the antiproliferative effect of PAS, tested as monotreatment (PAS+BIM: 90.1% ± 5.7 vs. CTR, PAS: 77.1% ± 3.2 vs. CTR; PAS+BIM vs. PAS *p* < 0.05) (Figure 5A).

Moreover, BIM-23627 alone (10^−8^ M concentration) did not affect intracellular cAMP levels in IBMX-treated cells (BIM: 97.3% ± 11.3 vs. CTR). However, when tested at equimolar concentration (10^−8^ M), BIM-23627 significantly impaired the inhibitory effect on cAMP accumulation induced by both OCT (OCT+BIM: 85.6% ± 12.2 vs. CTR, OCT: 37.9% ± 3.7 vs. CTR; OCT+BIM vs. OCT *p* < 0.001) and PAS (PAS+BIM: 97.6% ± 3.1 vs. CTR, PAS: 40.3% ± 6.4 vs. CTR; PAS+BIM vs. PAS *p* < 0.001) (Figure 5B).

### 3.6. OCT- and PAS-Induced Internalization of SST_2_ and SST_5_ in GH4C1 Cells

**Figure 6:**
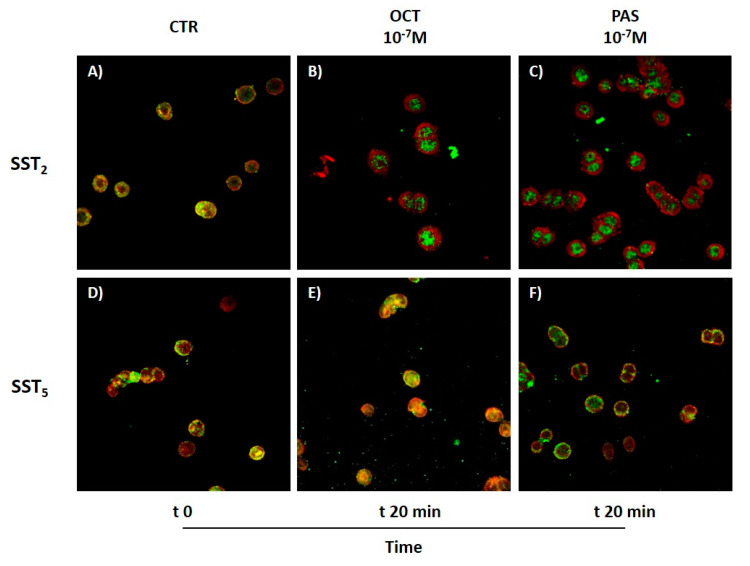
Effect of OCT and PAS treatment (10^−7^ M for 20 min) on SST_2_ and SST_5_ internalization in GH4C1 cells. Red fluorescence, DiI-stained cell membrane; green fluorescence, SST. (CTR, control; OCT, octreotide; PAS, pasireotide). Magnification: 40×.

To provide further insights into the biological mechanisms mediating the effects of OCT and PAS in our cell model, we investigated SST_2_ and SST_5_ activation following SRL treatment (10^−7^ M concentration, 20-min incubation), assessing the ligand-induced receptor internalization by immunocytofluorescence. Before drug exposure, GH4C1 cells were labeled with the red fluorescent lipophilic dye DiI to recognize cell membrane. Untreated cells showed a high level of co-localization between the receptors (green fluorescence) and the cell membrane (red fluorescence), resulting in a yellow fluorescent signal, as expected for resting G protein-coupled receptors (Figure 6A,D). Upon treatment with OCT and PAS, SST_2_ was almost completely internalized, suggesting that both compounds are powerful activators of this receptor subtype in GH4C1 cells (Figure 6B,C). Conversely, a completely different scenario was observed for SST_5_. Indeed, after 20-min treatment, this receptor subtype was mainly detected on cell membrane, thus laying for a faster internalization and recycling, or for a lack of activation after OCT and PAS binding (Figure 6E,F).

## 4. Discussion

In the present study, we described for the first time the effects of the combined treatment with OCT and PAS on GH secretion in human somatotroph primary cell cultures. We observed that, in consecutive tumor samples collected from acromegaly patients, the inhibitory activity of OCT, PAS and OCT+PAS was superimposable (all drugs tested at 10^−8^ M concentration, for 72 h).

Interestingly, we found that the antisecretory effect of the two compounds (tested alone or in combination) was significantly and directly correlated with the expression of SST_2_ (but not of SST_5_), when evaluated by immunohistochemistry performed in paraffin-embedded tissues.

Previous in vitro studies already highlighted SST_2_ as the main target for the PAS-induced inhibition of hormone secretion in somatotroph tumor cells [10,18,19,41]. Of note, Hofland and colleagues described a direct and significant correlation between SST_2_ mRNA expression and both OCT and PAS antisecretory activity [10]. In line with these findings, more recently our group confirmed that SST_2_ mRNA expression significantly correlated with the efficacy of OCT to reduce in vitro GH secretion, and a slight trend for linear correlation was observed for PAS as well [19]. Furthermore, in the same study we observed that the percentage of GH secretion decrease induced by OCT and PAS treatment was strongly and directly correlated when performing a pairwise comparison of different cell cultures (r = 0.829, *p* < 0.0001) [19]. It is noteworthy that this latest finding has been confirmed in the present manuscript (r = 0.85, *p* = 0.001).

As mentioned above, in the present study we find a significant correlation between the antisecretory effect of both OCT and PAS with SST_2_ protein expression. In this light, since the tested ligands directly interact with receptor proteins expressed at membrane level, these results strengthen the previous observations based on the mRNA expression of this target receptor.

Anyhow, a major finding of our study is the lack of any additive and/or synergistic effect of the combination treatment OCT+PAS in inhibiting in vitro GH secretion from human somatotroph tumor cells.

Early preclinical studies showed that the combination of SST_2_- and SST_5_-preferential agonists can act synergistically in the inhibition of GH secretion in both GHRH-stimulated human fetal pituitary cells, as well as in somatomammotroph pituitary tumor cells [9,42]. Therefore, based on the high binding affinity of PAS for SST_5_ (occurring in the subnanomolar range) [16], and the well described role of SST_5_ in the biological effects of PAS in other pituitary tumors (e.g., corticotroph tumors) [43], a potential advantage in combining OCT and PAS in the treatment of somatotroph tumors claimed to be investigated.

Interestingly, our results suggest that the in vitro antisecretory effect of both OCT and PAS is mediated by the activation of the same membrane receptor (namely, SST_2_). This finding discourages the use of a combined treatment with first- and second-generation SRLs in clinical studies aimed to increase the efficacy of the single compounds in the inhibition of GH secretion in acromegaly patients. On the other hand, if patients may benefit from the combined treatment OCT+PAS either in terms of circulating IGF-1 reduction, or for lowering PAS-induced glucose imbalance (as hypothesized by other authors) [44,45], cannot be clarified using our experimental setting.

Furthermore, the lack of additive and/or synergistic effects of the combination OCT+PAS has been confirmed in rat GH4C1 cells, a well-established model of somatotroph tumors. In particular, this immortalized cell line has been used to evaluate the impact of SRL combination therapy on cell proliferation (primary cultures of human somatotroph cells usually display a very low proliferation rate in vitro) [46], as well as on the modulation of pivotal intracellular mechanism involved in SST downstream, such as cAMP accumulation and [Ca^2+^]_i_ levels. In detail, we observed that OCT and PAS decreased GH4C1 cell proliferation at similar extent (about −25% vs. control), in line with previous observations [47,48,49]. Of note, their combination was not more superior than single treatment with both compounds, again suggesting that the same biological mechanism underlies the effects of both OCT and PAS on cell proliferation, as well. Interestingly, measuring the CI of the effects of the two drugs, we observed a moderate antagonism between OCT and PAS, which supports the role of SST_2_ as the main target for both drugs. Indeed, previous studies identified PAS as a partial agonist of SST_2_, which could therefore slightly counteract OCT effects [23].

To further investigate the role of SST_2_ in driving the response to both drugs in our model, we co-incubated OCT- and PAS-treated cells with a selective SST_2_ antagonist (BIM-23627), already validated in previous preclinical studies [50,51,52,53]. Since we found that the addition of the SST_2_ antagonist almost completely counteracted the antiproliferative effect of both OCT and PAS, we speculate that, in our model, PAS impacts cell proliferation mainly via SST_2_ binding, as we also observed for OCT treatment.

This finding is of particular interest since SST_1_ and SST_3_, both bound with a relatively high affinity by PAS, have been previously reported as potential and promising targets for the control of cell growth in different pituitary tumor types, including somatotroph tumors [54,55,56,57,58,59]. Of note, with the present in vitro study we cannot exclude that PAS may exert different (and potentially stronger) effects, compared to first-generation SRLs, on the various indirect mechanisms involved in the control of pituitary tumor cell growth (e.g., inhibition of growth factors and angiogenesis) [48,60,61].

In line with the data observed for cell proliferation, we observed that the combination treatment OCT+PAS showed similar efficacy compared to monotreatment, in terms of both inhibition of cAMP accumulation and decrease of KCl-stimulated [Ca^2+^]_i_ increase. Furthermore, co-incubation with the SST_2_-selective antagonist reversed the inhibitory effect of OCT and PAS on cAMP.

These latest results strengthen our observations on the effect of OCT, PAS and their combination on GH secretion in somatotroph tumor primary cultures. Indeed, the cAMP pathway and the regulation of ion conductance to prevent [Ca^2+^]_i_ increase are crucial biological mechanisms involved in the antisecretory activity exerted by SRLs in somatotroph tumors [7,62,63].

Overall, the similar efficacy showed by OCT and PAS, the lack of additive or synergistic effect observed for the combined treatment, as well as the complete antagonism showed by BIM-23627 on both compounds, all lay for a predominant role of SST_2_ in mediating the biological activities of the two SRLs in the tested models.

In this context, we are aware that the use of a SST_5_ antagonist (alone and in combination with BIM-23627) would have been precious to further strength our findings. Unfortunately, since no SST_5_-antagonist is commercially available, we could not get this compound.

Finally, we investigated the SST_2_ and SST_5_ trafficking in GH4C1 cells, in order to highlight a potential ligand-specific role in the modulation of receptor dynamics. Indeed, previous studies carried out in transfected cell models (e.g., HEK 230 and CHO-K1 cells) or non-pituitary cells endogenously expressing SSTs (e.g., AR42J cell line) showed that PAS results in a peculiar SST_2_ tracking rate (namely, lower internalization), compared to both OCT and endogenous SRIF [24,25,53,64]. Since receptor internalization is driven by ligand-activated phosphorylation of C-tail Serine and Threonine residues, a reduced SST_2_ phosphorylation following PAS treatment, compared to both OCT and SRIF, has been described also in rat pituitary GH3 cells (a cognate cell line of GH4C1) [53]. In the present study, we show that both OCT and PAS treatments result in a robust internalization of SST_2_ in cultured GH4C1 cells, in contrast to the evidence of previous studies carried out in transfected cell lines, although using comparable drug concentration and observation timing [24,25]. However, our finding fits with the results of functional studies demonstrating a similar efficacy of OCT and PAS in GH4C1 cells, thus supporting the powerful activation of SST_2_ exerted by both compounds in this specific cell model.

Of note, our observation highlights that significant cell-type differences may occur when investigating SST phosphorylation, trafficking and signaling, as already pointed out by other authors [18,65,66]. Furthermore, species-specific characteristics of SST have been emphasized, including a different pattern of ligand-induced phosphorylation for rat SST_2_ compared its human counterpart [2,64]. Therefore, although our findings on GH4C1 cells are consistent with the observations obtained from human somatotroph tumor primary cultures, this limitation has to be taken into account. Unfortunately, to date no immortalized cell lines derived from human somatotroph tumors are available for preclinical studies.

## 5. Conclusions

In primary cultures of somatotroph tumors, the combination treatment OCT+PAS is superimposable to single agent treatment in reducing GH secretion. Accordingly, the same results are observed when investigating cell proliferation, cAMP accumulation and modulation of [Ca^2+^]_i_ in GH4C1 cells. The antisecretory effect of OCT and PAS in human primary cultures significantly correlates with SST_2_ expression on paraffin-embedded tumor tissues, while in GH4C1 cells co-incubation with a SST_2_-specific antagonist almost completely counteracts the effects of both SRLs.

Taken together all this evidence suggests that, in our experimental setting, OCT and PAS seem to act mainly via SST_2_, thus playing down the potential usefulness of the combination treatment OCT+PAS, when looking to the pituitary-targeted effects of the two drugs, at least in acromegaly.

## Figures and Tables

**Table 1:** General, clinical and biochemical characteristics of patients at time of surgery and histopathological information of the tumors.

	Patient Characteristics					Tumor Characteristics						
Patient No.	Sex, Age (Years)	GH (µg/L)	IGF-1 (µg/L)	IGF-1 (xULN)	Pre-SurgerySRLs	Tumor Size	Ki-67	Mitoses (n/10 HPF)	p53 (+Nuclei/10 HPF)	Proliferation Index ^a^	SST_2_ Protein (IRS)	SST_5_ Protein (IRS)
1	M, 54	2.7	454	2.02	Yes	micro	<3%	0/10	6/10	0	9	6
2	F, 54	8.4	453	1.90	Yes	macro	<3%	0/10	2/10	0	8	8
3	M, 49	10.9	394	1.53	No	micro	<3%	0/10	13/10	1	12	9
4	M, 54	13.9	509	2.50	No	macro	<3%	1/10	0/10	0	12	12
5	M, 39	62.0	989	4.10	No	macro	<3%	0/10	0/10	0	8	12
6	F, 52	15.2	833	3.30	No	macro	<3%	0/10	5/10	0	12	1
7	F, 21	51.1	858	2.30	No	macro	<3%	0/10	2/10	0	8	4
8	M, 16	11.8	562	0.93	Yes	macro	≥3%	3/10	13/10	3	4	1
9	M, 50	8.0	469	1.89	No	macro	<3%	0/10	4/10	0	12	8
10	M, 57	14.0	571	2.86	No	macro	≥3%	8/10	>10/10	3	6	4
11	F, 39	7.9	436	1.80	No	macro	<3%	2/10	9/10	0	6	1

Legend: F, female; M, male; ULN, upper limit of normal; SRLs, somatostatin receptor ligands; HPF, high power fields; IRS, immunoreactivity score; micro, microadenoma (maximum diameter < 10 mm); macro, macroadenoma (maximum diameter ≥ 10 mm). ^a^ Proliferation index based on the score proposed by Trouillas and colleagues [29]. Highly proliferative (aggressive) lesions are defined in presence of at least two of the three following criteria: Ki-67: ≥3% (formalin fixative), mitoses: *n* > 2/10 HPF, p53: positive (10 strongly positive nuclei/10 HPF).

## Data Availability

All data generated or analyzed in this study are included in this published article (and its supplementary information files).

## Supplementary Materials

The following are available online at https://www.mdpi.com/article/10.3390/cancers13081816/s1: Figure S1: Dose-response curves of SRLs on GH secretion and cell proliferation in somatotroph primary cultures and in GH4C1 cell line, respectively; Figure S2: Correlation between the inhibitory combination effect on GH secretion and monotreatment effects or SST_2/5_ protein expression in somatotroph tumors; Figure S3: Expression of SST mRNAs and proteins in GH4C1 cells.
