# Supplementary figures and images for: Octreotide and Pasireotide Combination Treatment in Somatotroph Tumor Cells: Predominant Role of SST2 in Mediating Ligand Effects
Source: Cancers (Basel). 2021 Apr 10;13(8):1816. doi: 10.3390/cancers13081816 (PMC8069349; doi:10.3390/cancers13081816)

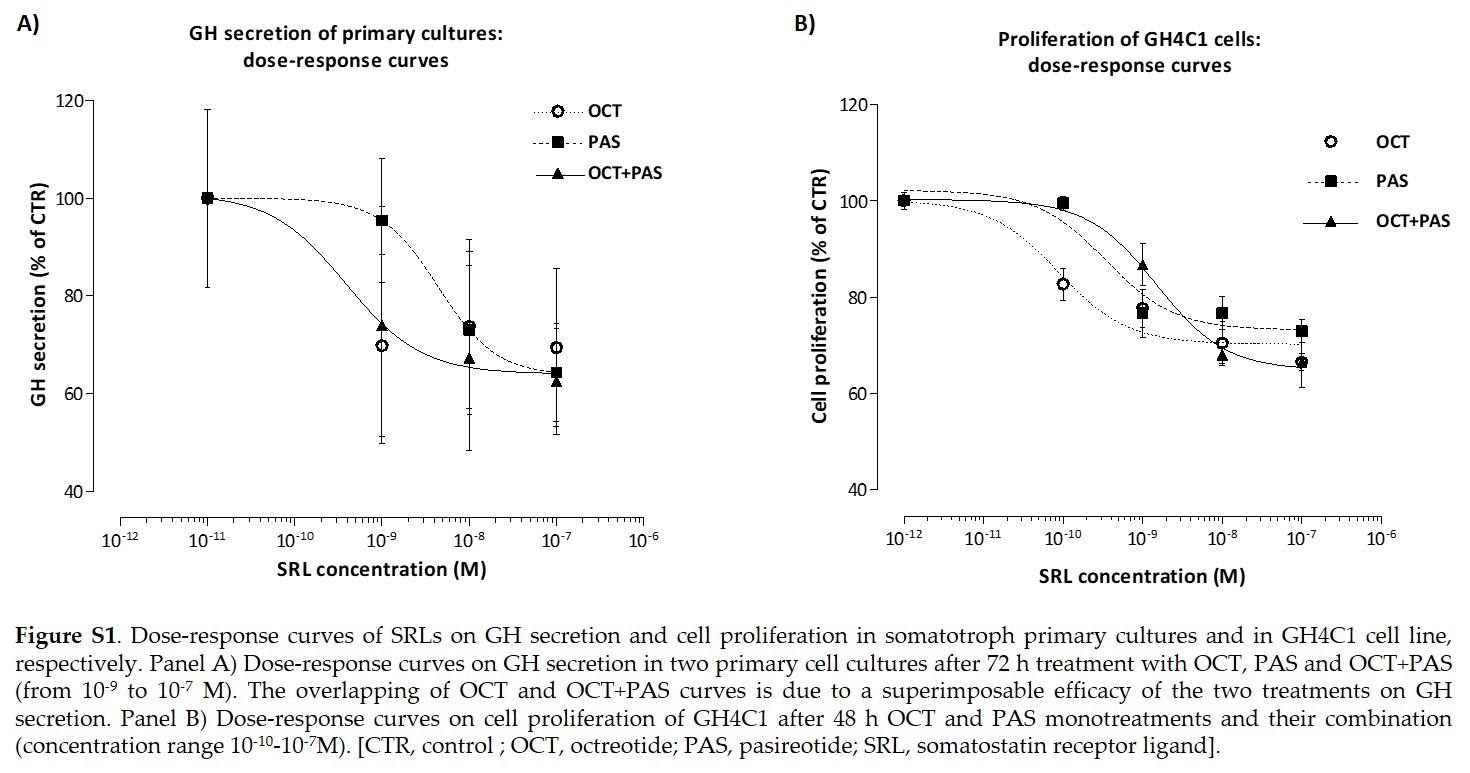

Supplement: Supplementary file 1 [file cancers-13-01816-s001.zip › Amarù J et al Supplementary Figures/FigureS1.jpg]

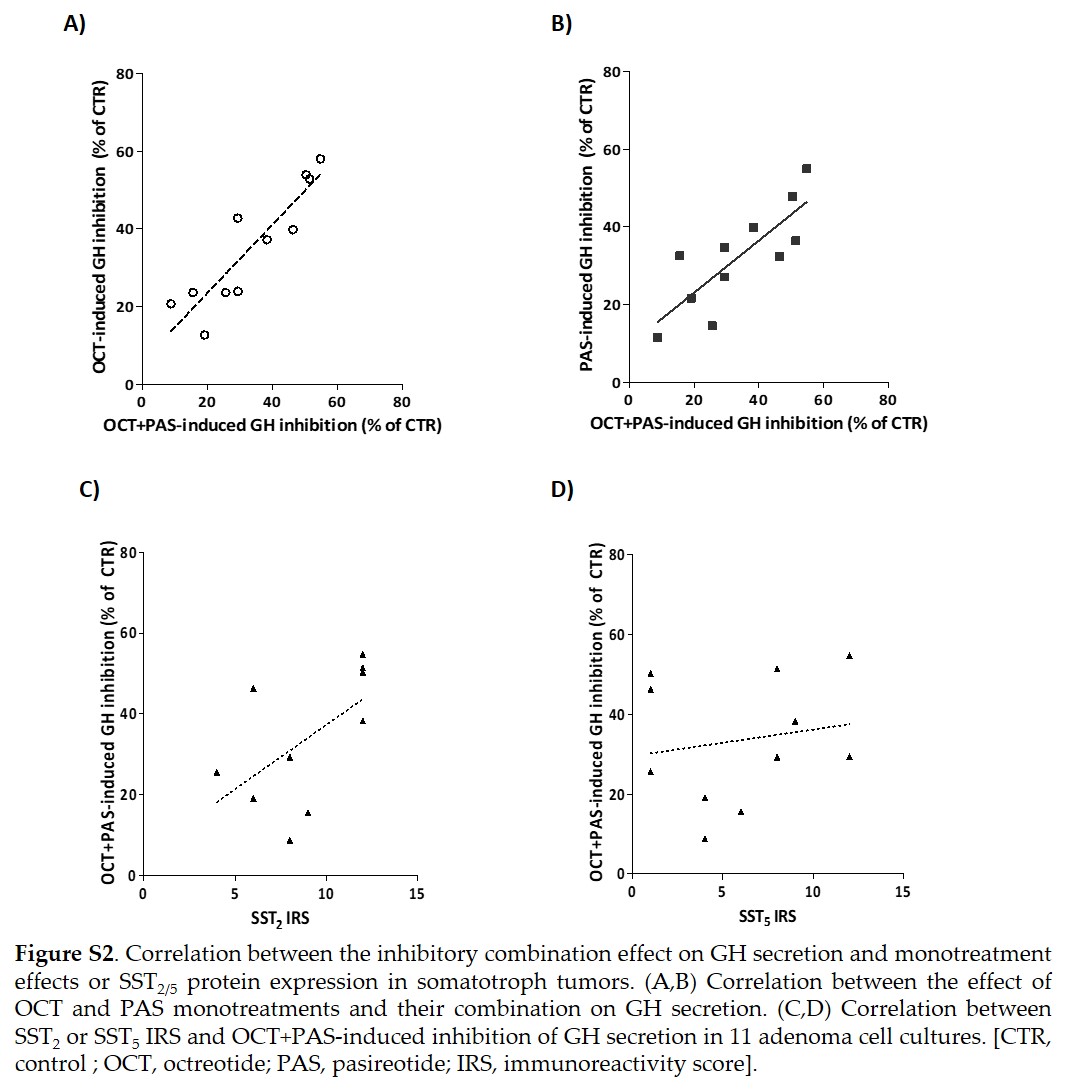

Supplement: Supplementary file 1 [file cancers-13-01816-s001.zip › Amarù J et al Supplementary Figures/FigureS2.jpg]

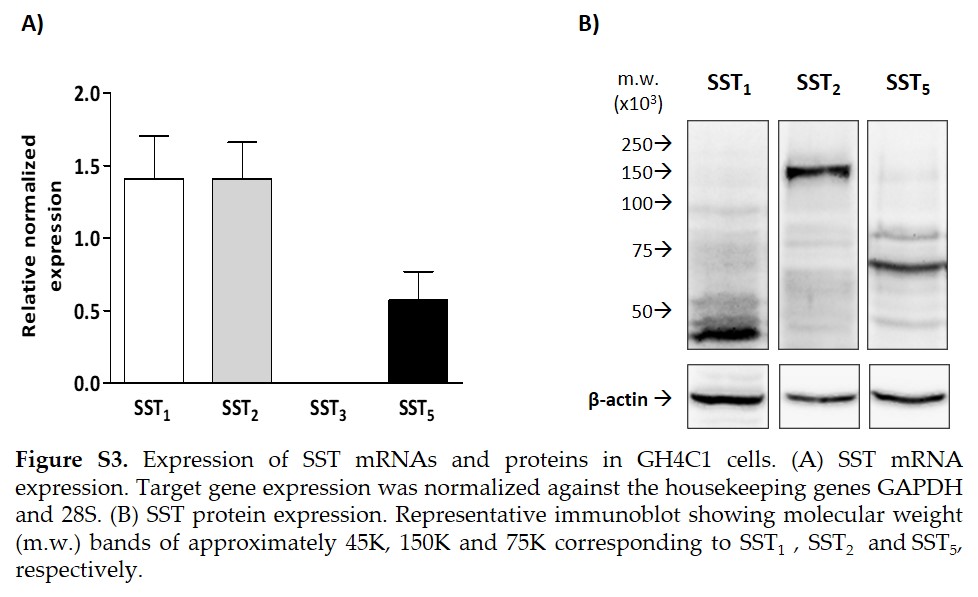

Supplement: Supplementary file 1 [file cancers-13-01816-s001.zip › Amarù J et al Supplementary Figures/FigureS3.jpg]
